# Supplementary material for: Seminoma and Embryonal Carcinoma Footprints Identified by Analysis of Integrated Genome-Wide Epigenetic and Expression Profiles of Germ Cell Cancer Cell Lines
Source: PLoS One. 2014 Jun 2;9(6):e98330. doi: 10.1371/journal.pone.0098330 (PMC4041891; doi:10.1371/journal.pone.0098330)
Supplement: File S1 — ZIP file containing DMRforPairs output for significant regions. Please start from the html files. (ZIP) [file pone.0098330.s008.zip › figures/28502.pdf]

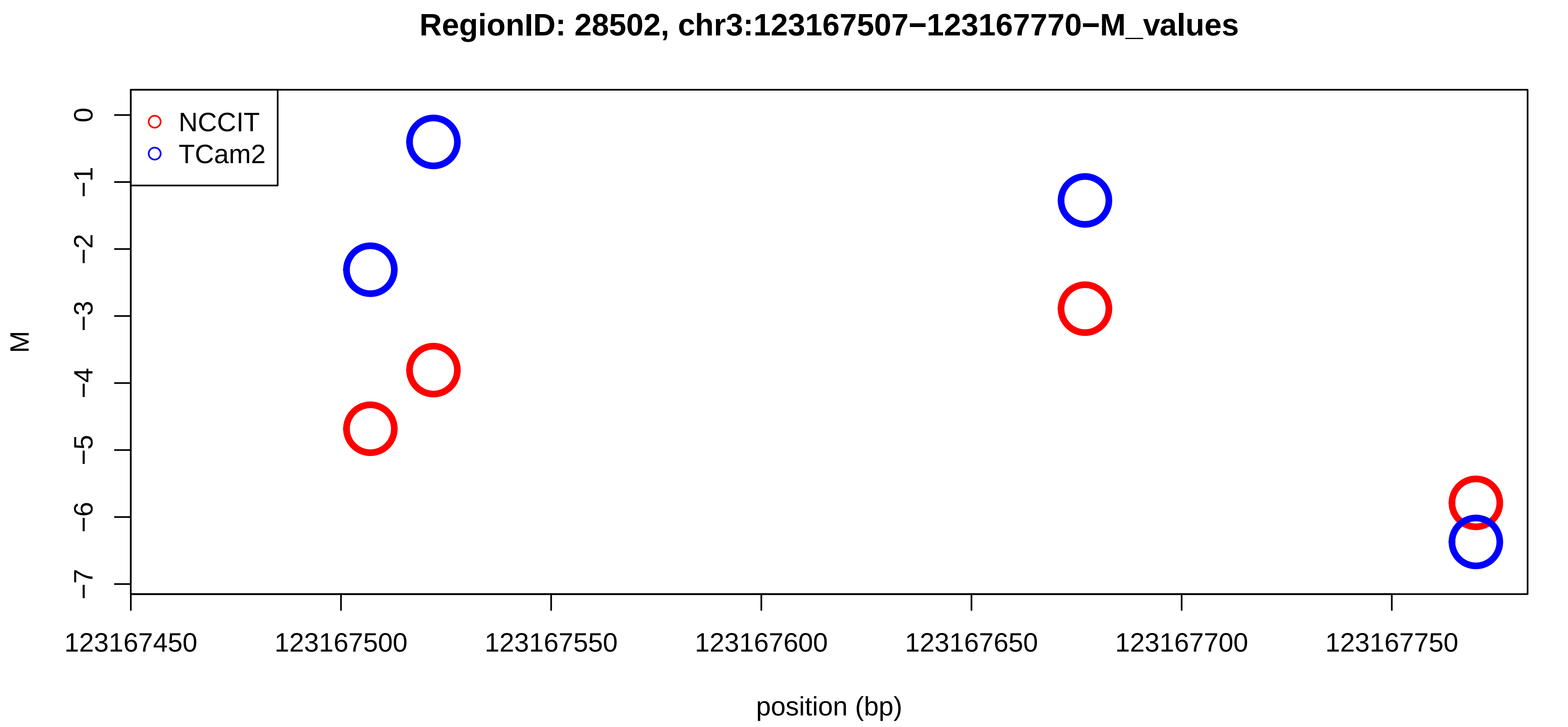
RegionID: 28502, chr3:123167507−123167770−M_values
NCCIT
TCam2
M
0
−1
−2
−3
−4
−5
−6
−7
123167450
123167500
123167550
123167600
123167650
123167700
123167750
position (bp)

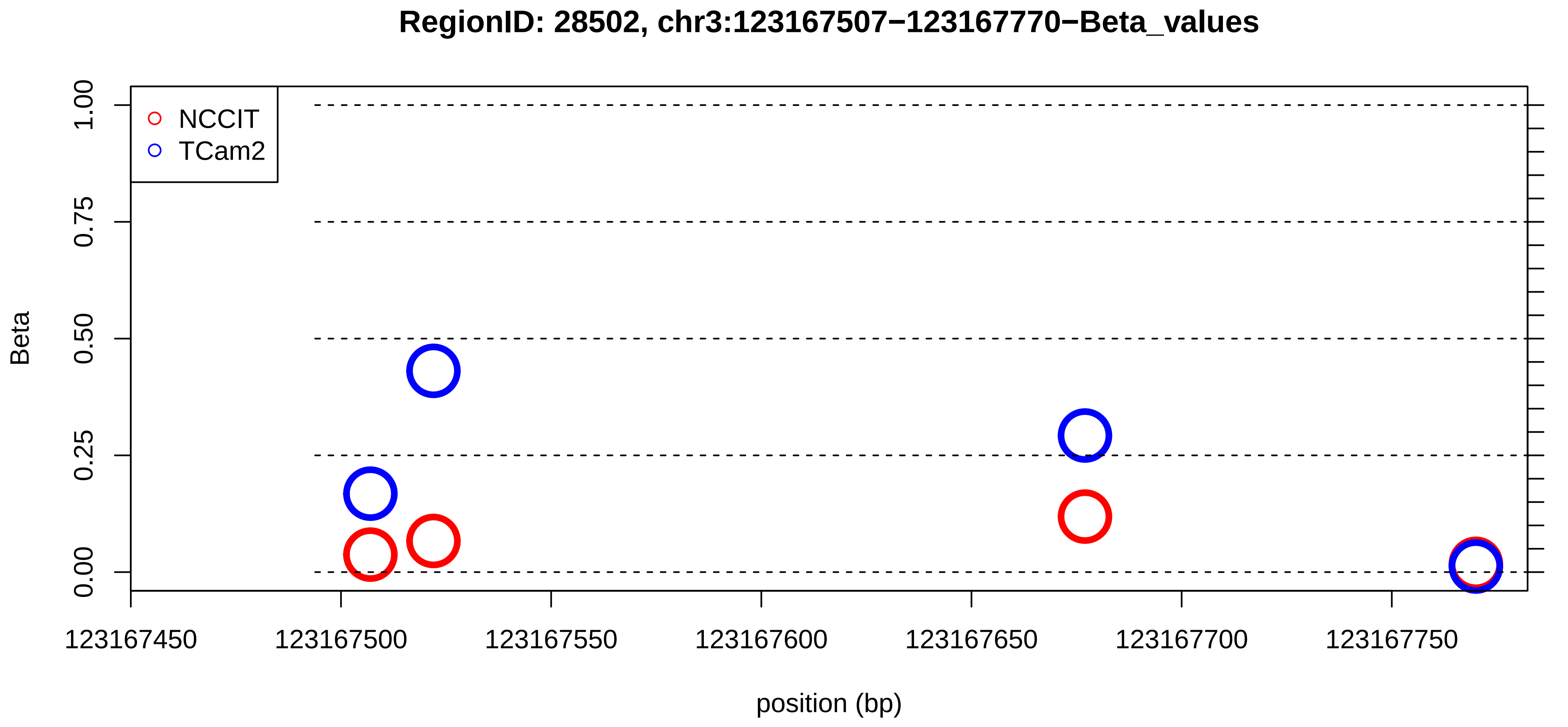
RegionID: 28502, chr3:123167507−123167770−Beta_values
NCCIT
TCam2
Beta
1.00
0.75
0.50
0.25
0.00
123167450
123167500
123167550
123167600
123167650
123167700
123167750
position (bp)
